# Social Isolation, Depressive Symptoms and Perceived Neighborhood Environment of Older Adults During COVID-19

**DOI:** 10.1093/geroni/igab046.2694

**Published:** 2021-12-17

**Authors:** Ke Li, Fengyan Tang

**Affiliations:** 1 University of Pittsburgh, Sandy Springs, Georgia, United States; 2 University of Pittsburgh, Pittsburgh, Pennsylvania, United States

## Abstract

Social isolation has been recognized as a social problem with negative effects on psychological well-being. Older adults are disproportionately affected by social isolation during the COVID-19 pandemic. Using data from the 2020 Health and Retirement Study COVID-19 Project, this study examined the relationship between social isolation and depressive symptoms among two groups of respondents differentiated by whether themselves or their social relationships were diagnosed with COVID-19. This study also explored the moderating role of perceived neighborhood environment. Depressive symptoms were measured using the eight-item CES-D. The index of social isolation was generated using five indicators, including living alone, no social participation, and less than monthly contact with children, family members, and friends. The moderator assessed two aspects of the neighborhood environment, including physical disorder and social cohesion. The results of bivariate analyses showed that respondents who were affected by COVID-19 were younger, more likely to be female, Hispanic, and Non-Hispanic Black, and with lower levels of social isolation. The results of multiple regression analyses indicated that social isolation was associated with more depressive symptoms, but this relationship was found to be only significant among respondents who were affected by COVID-19. Perceived neighborhood environment significantly moderated the relationship, as the effect of social isolation on depressive symptoms was stronger for respondents with more neighborhood physical disorders and less social cohesion. This study has implications for practice and policy, in that it underscored the importance of enacting strategies to improve the neighborhood environment, particularly for socially isolated older adults during the COVID-19.

